# Oleoylethanolamide Reduces Hepatic Oxidative Stress and Endoplasmic Reticulum Stress in High-Fat Diet-Fed Rats

**DOI:** 10.3390/antiox10081289

**Published:** 2021-08-14

**Authors:** Anna Maria Giudetti, Daniele Vergara, Serena Longo, Marzia Friuli, Barbara Eramo, Stefano Tacconi, Marco Fidaleo, Luciana Dini, Adele Romano, Silvana Gaetani

**Affiliations:** 1 Department of Biological and Environmental Sciences and Technologies, University of Salento, Via Prov.le Lecce-Monteroni, 73100 Lecce, Italy; daniele.vergara@unisalento.it (D.V.); serena.longo@unisalento.it (S.L.); stefano.tacconi@unisalento.it (S.T.); 2 Department of Physiology and Pharmacology “V. Erspamer”, Sapienza University of Rome, P.le Aldo Moro 5, 00185 Rome, Italy; marzia.friuli@uniroma1.it (M.F.); barbara.eramo@uniroma1.it (B.E.); adele.romano@uniroma1.it (A.R.); 3 Department of Biology and Biotechnology “C. Darwin”, Sapienza University of Rome, P.le Aldo Moro 5, 00185 Rome, Italy; marco.fidaleo@uniroma1.it (M.F.); luciana.dini@uniroma1.it (L.D.); 4 Research Center for Nanotechnology for Engineering of Sapienza (CNIS), Sapienza University of Rome, P.le Aldo Moro 5, 00185 Rome, Italy

**Keywords:** oleoylethanolamide, nuclear factor erythroid-derived 2-related factor 1 (Nrf1), nuclear factor erythroid-derived 2-related factor 2 (Nrf2), oxidative stress, diet-induced obesity, non-alcoholic fatty liver

## Abstract

Long-term high-fat diet (HFD) consumption can cause weight gain and obesity, two conditions often associated with hepatic non-alcoholic fatty liver and oxidative stress. Oleoylethanolamide (OEA), a lipid compound produced by the intestine from oleic acid, has been associated with different beneficial effects in diet-induced obesity and hepatic steatosis. However, the role of OEA on hepatic oxidative stress has not been fully elucidated. In this study, we used a model of diet-induced obesity to study the possible antioxidant effect of OEA in the liver. In this model rats with free access to an HFD for 77 days developed obesity, steatosis, and hepatic oxidative stress, as compared to rats consuming a low-fat diet for the same period. Several parameters associated with oxidative stress were then measured after two weeks of OEA administration to diet-induced obese rats. We showed that OEA reduced, compared to HFD-fed rats, obesity, steatosis, and the plasma level of triacylglycerols and transaminases. Moreover, OEA decreased the amount of malondialdehyde and carbonylated proteins and restored the activity of antioxidant enzymes superoxide dismutase, catalase, and glutathione peroxidase, which decreased in the liver of HFD-fed rats. OEA had also an improving effect on parameters linked to endoplasmic reticulum stress, thus demonstrating a role in the homeostatic control of protein folding. Finally, we reported that OEA differently regulated the expression of two transcription factors involved in the control of lipid metabolism and antioxidant genes, namely nuclear factor erythroid-derived 2-related factor 1 (Nrf1) and Nrf2, thus suggesting, for the first time, new targets of the protective effect of OEA in the liver.

## 1. Introduction

It is now widely accepted that lifestyle plays a fundamental role in maintaining a healthy status. Therefore, increased intake of high-palatable energy-dense foods, rich in sugars and lipids, predisposes the organism in the long-term to numerous metabolic diseases.

Obesity is characterized by an increase in body fat, caused by an energy imbalance, especially when consuming fat-rich diets. High-fat consumption, typical of the so-called Western diets, has been associated with insulin resistance, dyslipidemia, and metabolic/cardiovascular diseases [1,2,3]. Moreover, high-fat diet (HFD)-associated obesity is very common in patients affected by non-alcoholic fatty liver disease (NAFLD), which represents one of the most frequent causes of liver disease, particularly in Western countries [1,2,3].

NAFLD is characterized by excessive hepatic lipid accumulation [4] and is recognized as the typical hepatic manifestation of metabolic syndrome [5]. Several lines of evidence suggest that a plethora of dysfunctional processes occur during the progression of NAFLD including inflammation, mitochondrial and endoplasmic reticulum (ER) impairment and oxidative stress [6,7,8,9].

Oxidative stress is characterized by increased production of reactive oxygen species (ROS) and/or reduction in the antioxidant body defenses. Excessive levels of ROS can damage different cellular components, including protein, membrane lipids, and nucleic acids, thus affecting the whole body [10]. The main sites of ROS production comprise the mitochondrial electron transport chain, ER, peroxisomes and enzymatic sources such as nicotinamide adenine dinucleotide phosphate oxidase and uncoupled endothelial nitric oxide synthase [11].

The pathways for ROS production and oxidative stress have been reported to be upregulated in the liver of mice consuming an HFD, and this event precedes the onset of insulin resistance [12]. Moreover, both animal and human studies have reported that long-term HFD feeding leads to increased oxidative stress and dysfunctional mitochondria in many organs [13,14,15,16] and induces ER stress in vitro [6,7,8,9]. The functional crosstalk between oxidative stress and ER stress is well described. Alterations in the protein folding process and enhanced production of misfolded proteins can exacerbate oxidative stress [17].

Therefore, antioxidants that preferentially remove oxidative stress may have therapeutic applications in ROS-induced chronic alterations.

Oleoylethanolamide (OEA) is a naturally occurring bioactive lipid belonging to the N-acylethanolamides family. In the last two decades, this molecule has received great attention thanks to its biological properties [18,19]. In the small intestine of different species, including rats and mice, OEA can be synthesized from diet-derived oleic acid [20,21,22]. The OEA synthesis is controlled, among others, by the membrane protein CD36, a multiligand class B scavenger receptor located on cell surface lipid rafts, which acts as a biosensor for food-derived oleic acid [23,24].

OEA has been demonstrated to exert a plethora of protective effects including anti-obesity, anti-inflammatory, and antioxidant properties thus supporting its potential use for the treatment of obesity and eating-related disorders [18,25,26]. In fact, as a drug, OEA reduces food intake and body weight gain [20,27,28] in both lean and obese rodents and reduces lipopolysaccharide-induced liver injury in mice [29]. Moreover, it has been demonstrated that OEA reduces lipid synthesis and lipoprotein secretion in hepatocytes [30] and improves HFD-induced liver steatosis in rats [31] and humans [32,33]. Recently, we demonstrated that OEA decreases hepatic lipid accumulation through a peroxisome proliferator-activated receptor γ (PPARγ)-mediated inhibition of hepatic fatty acid uptake and triacylglycerol synthesis [34].

Although several studies have investigated both behaviourally and neurochemically [18,19,20,21,22,23,24,25,26] the anti-obesity properties exerted by OEA, the molecular mechanism underlying the protective role of this compound on HFD-induced liver damages remains largely unknown.

In the present study, we exploited a rat model of diet-induced obesity to evaluate the potential beneficial effect of OEA on HFD-induced hepatic oxidative stress, by analyzing the activity of antioxidant enzymes and expression of proteins involved in oxidative stress and ER homeostasis. Moreover, the expression of the nuclear factor erythroid-derived 2-related factor 1 (Nrf1) and Nrf2, proteins involved in the regulation of oxidative stress, was also evaluated, to speculate a functional hypothesis on the potential antioxidant effect of OEA in the liver of HFD-fed rats.

Our results suggest that OEA reduces fatty liver, liver damage, oxidative stress, and ER stress in the liver of HFD-fed rats through a mechanism that differentially involves Nrf1 and Nrf2 thus expanding the already known anti-obesity effects of OEA to new targets.

## 2. Materials and Methods

### 2.1. Animals, Diet, and Chronic Treatments

**Figure 1:**
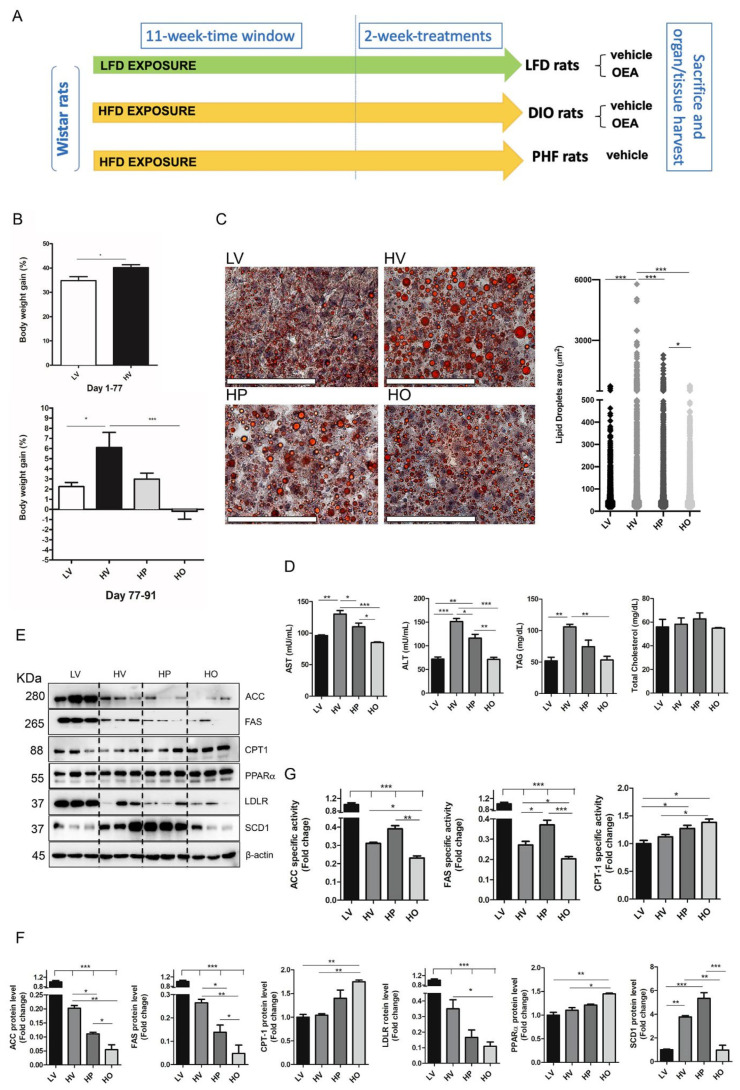
OEA ameliorates parameters of liver steatosis. (**A**) Schematic representation of rat treatments (*n* = 20). (**B**) Body weight gain was measured after the pre-experimental period (77 days) and at the end of the experimental period (77–91 days). (**C**) Representative microscopic observations of the Oil red-*O*-stained lipid droplets and relative quantification from low-fat (LV) (*n* = 5), high fat (HV) (*n* = 5), pair feeding (HP) (*n* = 5) and HV+OEA (HO) (*n* = 5) rats. Scale bar = 50 μm. (**D**) Plasma levels of aspartate aminotransferase (AST), alanine aminotransferase (ALT), triacylglycerols (TAG) and total cholesterol in the four experimental groups. Values in the histogram represent the mean ±  SEM (*n* = 4). (**E**) Representative immunoblots for acetyl-CoA carboxylase (ACC), fatty acid synthase (FAS), carnitine palmitoyltransferase-1 (CPT-1), peroxisome proliferator-activated receptor α (PPARα), low-density lipoprotein receptor (LDLR), and stearoyl-CoA desaturase 1 (SCD1); β-actin was used as a loading control. (**F**) Quantification of protein expression. Values in the histogram represent the mean ±  SEM (*n* = 3) and are expressed as fold change relative to LV. (**G**) Specific activity of ACC, FAS, and CPT-1. Activities values are expressed as fold change relative to LV. Values are the mean ± SEM of five different samples. * *p* < 0.05; ** *p* < 0.005; *** *p* < 0.001.

Adult male Wistar-Han rats (250–300 g at the beginning of the study) were housed in single cages under controlled conditions of temperature and humidity (T = 22 ± 2 °C; 60% of relative humidity) and were kept on a 12 h light/dark cycle. The study scheme is presented in Figure 1A. In particular, a total of 20 rats were divided into two groups, one receiving the low-fat diet (LV) (*n* = 5) and the other fed on an HFD (*n* = 15), for a total of 77 days. Both low-fat and high-fat diets were from Research Diet (New Brunswick, NJ, USA; HFD, Code: D12492; https://researchdiets.com/formulas/d12450B (accessed on 7 June 2021); LV, Code: D12450B; https://researchdiets.com/formulas/d12450B (accessed on 7 June 2021)). The HFD-fed group of animals was then divided into three experimental groups and subjected for two weeks to the following treatments: one group continued to receive the HFD ad libitum and received a daily intraperitoneal (i.p.) administration of vehicle (HV) (*n* = 5), the second group was fed ad libitum with the HFD and daily treated with OEA 10 mg/kg, i.p. (HO) (*n* = 5), and the third group was i.p. injected with a vehicle (2 mL/kg) and provided, each day, with the average amount of food eaten by matched, free-feeding OEA-treated animals (HP) (*n* = 5). This pair-feeding procedure was undertaken to overcome the problem related to the reduction of food intake induced by OEA [35], avoiding the uncertainty that observed metabolic effects could be not due to the direct action of OEA but rather to its anorexic properties. All groups had free access to water. The treatments lasted for a total of 2 weeks, while animals of the LV group received ad libitum low-fat diet and were daily treated with the vehicle (2 mL/kg, i.p.). Both OEA and vehicle solutions were freshly prepared on each test day and administered about 10 min before dark onset by following our previous protocols [35,36,37]. OEA was synthetized in the laboratory [38] and administered by i.p. injection at the dosage of 10 mg/kg in a vehicle of saline/polyethylene glycol/Tween 80 (90/5/5, *v*/*v*/*v*). Food intake and animal body weights were daily monitored.

At the end of the 2-week treatments, animals were sacrificed, blood was collected in microcentrifuge tubes containing EDTA and after centrifuge plasma was recovered and stored at −80 °C until processed; livers were also immediately collected, washed in ice-cold phosphate-buffered saline, snap-frozen in 2-metylbutane (−60 °C), and stored at −80 °C until analysed. All experiments were carried out under the European directive 2010/63/UE governing animal welfare and according to the Italian Ministry of Health guidelines for the care and use of laboratory animals.

### 2.2. Plasma Parameter Assays

Plasma triacylglycerol (cat. no. TR210, Randox, Crumlin, UK), total cholesterol (cat. no. CH200, Randox), aspartate aminotransferase (AST, cat. no. MAK055, Sigma-Aldrich, Milan, Italy), and alanine aminotransferase (ALT, cat. no. MAKO52, Sigma-Aldrich) analyses were performed using commercially available kits, according to the manufacturer’s instructions.

### 2.3. Oil Red-O Staining of Lipid Droplets and Quantification

Fresh snap-frozen liver samples were cutted on a cryostat in 20-μm-thick serial sections and stored at −80 °C until processed for Oil Red-O staining. On the day of the experiment, liver sections were fixed in 4% paraformaldehyde in PBS pH 7.4 for 5 min at room temperature; after several washes in distilled water sections were stained with Oil Red-O solution at 5% in isopropyl alcohol for 1 h. Sections were then rinsed with distilled water, washed with 10% isopropanol, and counterstained with hematoxylin for 30 s. Sections were then visualized and photographed by EVOS FL Auto Cell Imaging System (Thermo Fisher Scientific, Waltham, MA, USA) with PlanApo N 60 X/1.42 oil (Olympus, Shinjuku, Tokyo, Japan) at ×40 magnification. Lipid droplet average size was determined as reported in [39] through ImageJ Software (Version 1.5Oi, NIH, Bethesda, MD, USA). Measures were obtained by analyzing at least 50 sections for each group in three independent experiments.

### 2.4. Assay of Enzymatic Activities Related to Fatty Acid Synthesis and Oxidation

Acetyl-CoA carboxylase (ACC) and fatty acid synthase (FAS) activities were determined in liver homogenate, as previously reported [40]. To determine ACC activity, we measured the incorporation of [1-^14^C]acetyl-CoA into fatty acids in a coupled assay with the FAS reaction. The reaction was carried out at 37 °C for 8 min. To determine FAS activity, malonyl-CoA was included in the ACC assay mixture, while ATP, butyryl-CoA, and FAS were omitted. The assay measurement ended after 10 min. Carnitine palmitoyltransferase-1 (CPT-1) activity was measured as the incorporation of radiolabelled carnitine into acylcarnitine, as previously reported [41].

### 2.5. Hepatic Antioxidant Enzymatic Activities

The glutathione peroxidase (GPx) assay was performed following the instructions reported in Cayman’s GPx assay kit (cat. no. 703102, Cayman Chemical, Ann Arbor, MI, USA). The activity was measured indirectly by a coupled reaction with glutathione reductase (GR). Oxidized glutathione produced upon reduction of hydroperoxide by GPx was recycled to its reduced state by GR and nicotinamide adenine dinucleotide phosphate. The change in absorbance at 340 nm was monitored for 3 min. A blank with all ingredients except for the sample was also included in the procedure. Specific activity was calculated as U/mg protein.

Superoxide Dismutase (SOD) activity was assayed by SOD assay Kit (cat. no. 19160, Sigma-Aldrich), which utilized a tetrazolium salt for detection of superoxide radicals generated by xanthine oxidase and hypoxanthine. SOD activity was calculated using the equation obtained from the linear regression of a standard curve built in the same experimental conditions.

Catalase activity was assayed by a catalase assay kit (cat. no. 219265, Sigma-Aldrich) that uses a reaction between the catalase present in the sample and hydrogen peroxide (H_2_O_2_) to produce water and oxygen. The unconverted H_2_O_2_ reacts with a probe to generate a product that can be measured colorimetrically at 540 nm.

### 2.6. Hepatic Oxidative Stress Parameters

H_2_O_2_ was measured by Amplex^®^ Red Hydrogen Peroxide/Peroxidase Assay Kit (cat. no. A22188, Thermo Fisher Scientific). The oxidation product of 1:1 stoichiometric reaction between Amplex^®^ Red reagent and H_2_O_2_ was spectrophotometrically measured at 571 nm.

For the malondialdehyde (MDA) assay, liver tissues were homogenized in the lysis buffer. MDA levels were measured by Lipid Peroxidation MDA assay kit according to manufacturer’s instructions (cat. no. MAK085, Sigma-Aldrich).

Oxidative damage of proteins was determined by quantifying carbonyl groups by OxyBlotTM Protein Oxidation Detection Kit (cat. no. S7150, Sigma-Aldrich), according to the manufacturer’s recommendations. Briefly, carbonyl groups were derivatized with 2,4-dinitrophenylhydrazine to obtain 2,4-dinitrophenylhydrazone (DNP) products. Non-derivatized samples were used as negative controls. Both derivatized and non-derivatized samples were separated by SDS-PAGE and transferred onto polyvinylidene difluoride membranes. Membranes were blocked with phosphate-buffered saline supplemented with 1% bovine serum albumin (BSA), 0.1% Tween 20 and incubated with a primary antibody (1:150) against DNP-modified carbonyl groups. Protein-antibody complexes were visualized using an ECL Advance Western Blotting Detection Kit (GE Healthcare, Chicago, IL, USA).

### 2.7. Western Blot Analysis

Proteins were extracted from the liver homogenate using RIPA lysis buffer (cat. no. 9806, Cell Signaling, Danvers, MA, USA). Total protein levels of the lysate were determined using the Bradford method (BIORAD, Hercules, CA, USA). After boiling for 5 min, proteins were loaded and separated by SDS-polyacrylamide gel. The samples were then transferred to a nitrocellulose membrane (Bio-Rad Laboratories) and blocked at room temperature for 1 h using 5% (*w*/*v*) non-fat milk in TBS-Tris buffer (Tris-buffered saline (TBS) plus 0.5% (*v*/*v*) Tween-20, TTBS). The membranes were incubated overnight at 4 °C with primary antibodies against ACC (cat. no. 3676, Rabbit 1:1000, Cell Signaling), FAS (cat. no. 3180, Rabbit 1:1000, Cell Signaling), stearoyl-CoA desaturase 1 (SCD1) (cat. no. sc-58420, Mouse 1:1000, Santa Cruz, CA, USA), low-density lipoprotein receptor (LDLR) (cat. no. PAB8804, Rabbit 1:1000, Abnova, Milan, Italy), CPT-1 (cat. no. 12252S, Rabbit 1:500, Cell Signaling), PPARα (cat. no. sc-9000, Rabbit 1:1000, Santa Cruz), Nrf1 (cat. no. sc-28379, Mouse 1:1000, Santa Cruz), Nrf2 (cat. no. sc- 365949, Mouse 1:1000, Santa Cruz), PPARγ (cat. no. 2430S, Rabbit 1:1000, Cell Signaling), X-box-Binding Protein-1 (XBP1) (cat. no. sc-7160, Rabbit 1:1000, Santa Cruz), Activating Transcription Factor 6 (ATF6) (cat. no. sc-166659, Mouse 1:1000, Santa Cruz), and binding-immunoglobulin protein aka GRP-78 (BIP) (cat. no. 3177, Rabbit 1:1000, Cell Signaling). β-actin antibody (cat. no. 8457, Rabbit 1:1000, Cell Signaling), was used as a loading control. After washing with TTBS, the blots were incubated with peroxidase-conjugated secondary antibodies (cat no. A3687 and cat no. A3652, Sigma-Aldrich,) at 1:10,000 dilutions at room temperature for 1–2 h. The blots were then washed twice in TTBS. Western blotting analyses were performed using the Amersham ECL Advance Western Blotting Detection Kit (cat. no. RPN2106, GE Healthcare, Little Chalfont, UK). Densitometric analysis of immunoblots was performed using the Image Lab^TM^ Version 6.0.1 2017 software (Bio-Rad Laboratories, Inc.).

### 2.8. Isolation of Total RNA and RT-qPCR

About 50 mg of liver per sample was placed in a 1.5-mL tube and homogenized using micropestle. Immediately, TriFast reagent (EuroGold Trifast^TM^ kit, Euroclone, Milan, Italy) was added to samples and they were further disaggregated by vigorously pipetting. Total RNA was extracted in accord with the manufacturer’s instructions. NanoDrop and denaturing agarose gel electrophoresis were used to quantify mRNAs and check their integrity, respectively. The first-strand cDNA was obtained from 1 μg of RNA and a mix of random hexamer and oligo-dT by SensiFastTM cDNA Synthesis Kit (Aurogene, Rome, Italy). Synthesized cDNA corresponding to 50 ng of total RNA was used for each reaction of RT-qPCR using SensiFastTM SYBR Green Lo-ROX kit (Aurogene, Rome, Italy) and QuantStudio 3 Real-Time PCR Systems (Thermo Fisher Scientific) according to manufacturer’s instructions. The sequence of primers is as follows: Nqo1 (NAD(P)H Quinone Dehydrogenase 1) forward: GCCTACACGTATGCCACCAT; reverse: TGGACACCCTGCAGAGAGTA. Ho-1 (Heme oxygenase-1): forward: CTAAGACCGCCTTCCTGCTC; reverse: GCCTCTGGCGAAGAAACTCT. Gclc (Glutamate-Cysteine Ligase Catalytic Subunit): forward: CTGCAGAGGAGTACACGCTC; reverse: GTCCACGTCGACTTCCATGT. B2m (β-2-Microglobulin): forward: AATTCACACCCACCGAGACC; reverse: TCCATAGAGCTTGATTACATGTCTC. Sequences were designed for annealing flanking exons of specific target genes and for producing 80–120-bases long amplicons. Relative changes in gene expression were calculated according to the delta-delta Ct method using B2m as a reference gene and normalized to LV treatment.

### 2.9. In Vitro Studies with Hepatic Cells

The human hepatocellular carcinoma cell line Huh7 was maintained in Dulbecco’s minimum essential medium eagle (DMEM) low glucose with 10% fetal bovine serum, 100 U/mL penicillin, 100 μg/mL streptomycin, and 2 mM glutamine. Cells were cultured at 37 °C with 5% partial pressure of CO_2_ in a humidified atmosphere. To induce lipid droplet accumulation, cells were cultured with a mix of palmitate-oleate (free fatty acids, FFA, 1:2 molar ratio), at a final concentration of 500 µM. FFA were complexed to BSA, at a final molar ratio of FFA/BSA of ∼2:1, which is close to the ratio observed in human serum [42]. In detail, Huh7 cells were exposed to FFA for 48 h or exposed to FFA for 24 h and then to a combination of FFA and OEA for additional 24 h. OEA was dissolved in dimethyl sulfoxide (DMSO) at a concentration of 10 mM and used at the concentration of 10 μM. Control cells (CTR) were treated with BSA alone or in combination with OEA.

To visualize lipid droplet accumulation, cells were stained for 30 min with BODIPY 493/503 (cat. no. 25892, Cayman Chemical) and NucBlue™ Live ReadyProbes™ Reagent (33342, Hoechst) (cat. no. R37605, Thermo Fisher Scientific) followed by two washes with PBS and visualized with an EVOS FLoid Imaging System microscopy (Thermo Fisher Scientific). Fluorescence intensity was quantitated using Image J software (Version 1.53) and normalized respect to total number of nuclei. At the end of the incubation time, cells were scraped off and SOD activity and H_2_O_2_ amount were determined as above reported.

### 2.10. Statistical Analysis

Results are expressed as means ± standard error of the mean (SEM). Statistical differences were evaluated using GraphPad Prism version 8.3.0 for Windows (GraphPad Software, San Diego, CA, USA). The comparison was made using one-way analysis of variance (ANOVA) and Tukey post hoc analysis. Differences between groups were considered statistically significant for *p* < 0.05.

## 3. Results

### 3.1. OEA Inhibited the Increase of Body Weight and Prevented Hepatic Histopathological Changes of HFD-Fed Rats

We measured body weight gain at the end of the pre-experimental period (77 days) and after two weeks of treatment (Figure 1B). After 77 days of feeding, rat body weight in HFD was significantly increased, as compared to LV, thus indicating the establishment of the obesity rat model (Figure 1B).

At the end of the two-week-treatment period (91st day), HFD-fed rats maintained significantly higher body weight as compared to LV. Moreover, despite the similar energy intake between HP and HO groups, HP animals had a greater body weight gain than OEA-treated rats and did not show any significant difference from HV animals. Conversely, OEA significantly decreased body weight gain of HO rats, as compared to HV rats (Figure 1B), thus suggesting that OEA treatment reduced body weight gain through a mechanism that was independent from its anorexigenic effect.

Moreover, the results obtained from the histological analyses of liver sections conducted through the O-red oil staining protocol showed that HFD per se caused a significant increase in the amount of lipid stored as lipid droplets (HV vs. all the other groups, Figure 1C) and that such increase was completely abolished in both the HO and HP groups of rats, as compared to the HV group. Moreover, animals treated with OEA showed a further significant decrease in the mean particle size, compared to HP rats, thus suggesting that the fat accumulation of HFD-fed rats can be successfully decreased by OEA treatment, and this effect might not be the mere consequence of the reduced caloric intake induced by OEA. Consistent with the increased size of lipid droplets observed in the liver, we found that HV rats had a significant increase, compared with LV, in the plasma level of triacylglycerols, which was reversed by the OEA treatment (Figure 1D). No significant changes were, instead, measured, in the plasma level of total cholesterol. Furthermore, the plasma activity of AST and ALT, two key markers of liver damage, increased in HV and was significantly reduced in HO rats (Figure 1D).

### 3.2. OEA Reduced Hepatic Steatosis by Increasing Fatty Acid Oxidation and Decreasing Lipogenesis

To explore the mechanism by which OEA improves fatty liver disease, we determined the expression of key proteins involved in fatty acid synthesis and oxidation. In this respect, we measured the activity and expression of ACC and FAS, key enzymes involved in the fatty acid synthesis pathway, and PPARα and CPT-1, involved in the fatty acid oxidation process.

Western blotting analyses (Figure 1D,E) showed that, as expected, the expression of both ACC and FAS in the HV group was significantly lower compared to LV. Moreover, a significantly reduced expression in LDLR, involved in the transport of lipoproteins into the liver, after HFD feeding, was also detected. When compared to the HV group, HO rats showed significantly decreased ACC, FAS and LDLR expression (Figure 1D,E), with concomitant significant reductions of ACC and FAS activities (Figure 1F) that reached values significantly lower in rats treated with OEA, compared to both HV and HP groups. To note that both ACC and FAS expression and specific activities were lower in HO than in HP, thus indicating that these effects could be specifically attributed to OEA and not to its anorexigenic effect. Moreover, OEA administration to HFD-fed rats significantly increased both PPARα and CPT-1 expression (Figure 1D,E), with a concomitant up-regulation of CPT-1 activity (Figure 1F). Compared to both HV and HP groups of rats, those treated with OEA showed a significant decrease in the level of SCD1, a protein that plays an important role in hepatic lipid homeostasis [43] (Figure 1D,E).

These results suggested that OEA likely ameliorates NAFLD parameters by increasing hepatic fatty acid oxidation and decreasing lipid transport and fatty acid synthesis.

### 3.3. OEA Improved Hepatic Oxidative Stress Both In Vivo and In Vitro

**Figure 2:**
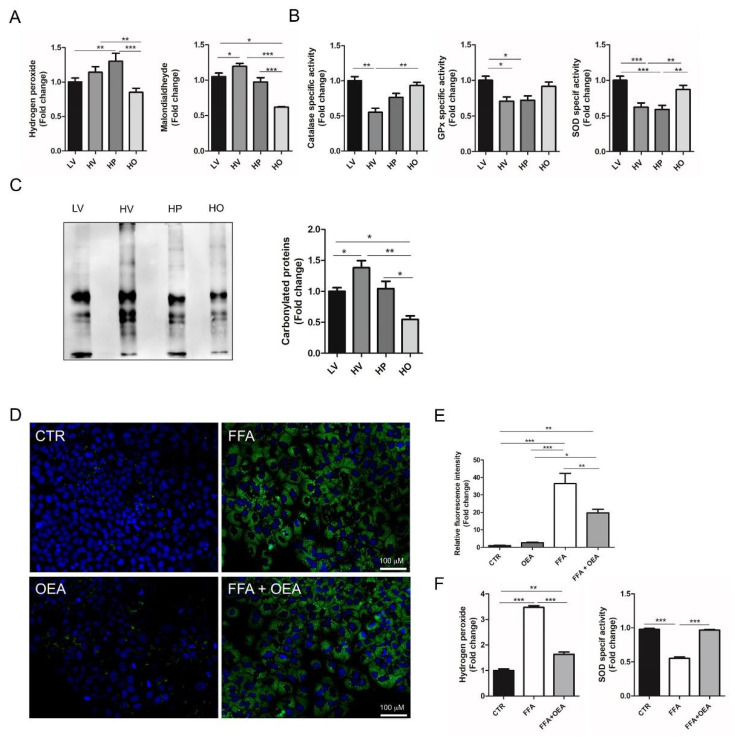
OEA modulated oxidative stress in vivo and in vitro. (**A**) Hydrogen peroxide and malondialdehyde measurements were done as reported in Materials and Methods. Values, expressed in fold change of LV, are the mean ± SEM of five different samples. (**B**) Catalase, glutathione peroxidase (GPx), and superoxide dismutase (SOD), specific activities were assayed in liver homogenates as reported in Materials and Methods. Values, expressed in fold change of LV, are the mean ± SEM of five different samples. (**C**) Representative immunoblot for carbonylated proteins and relative quantification. Values, expressed in fold change of LV, are the mean ± SEM of three different samples. (**D**) Representative images of Huh7 cells treated with BSA (CTR), BSA and OEA (OEA) or with a mix of palmitate-oleate (FFA) in BSA at 500 µM final concentration alone or in combination with OEA as described. Cells were stained with BODIPY 493/503 (lipid droplets, green), and Hoechst 33342 (nucleus, blue), scale bar is 100 μm. Fluorescence images were captured using an EVOS FLoid Imaging System. (**E**) Fluorescence intensity for each image was quantitated using Image J software and normalized respect to total number of nuclei. The fluorescence intensity, relative to CTR, is shown and expressed as mean ± SEM (*n* = 4). (**F**) Hydrogen peroxide and SOD activity were assayed in CTR, FFA, and FFA+OEA. Values, expressed in fold change of CTR, are the mean ± SEM of five different experiments. * *p* < 0.05; ** *p* < 0.005; *** *p* < 0.001.

To test the antioxidant effect of OEA in the liver of HFD-fed rats, we first measured selected parameters associated with oxidative stress in HV as compared to LV rats. Our data showed that HFD feeding induced greater hepatic H_2_O_2_ accumulation, as compared to the LV diet. HP groups of rats showed significantly increased levels of H_2_O_2_, compared to the LV group (Figure 2A).

One of the primary events during oxidative stress is the peroxidative damage to membrane lipids, which can be determined by their degradation product MDA. Indeed, an increased level of MDA was measured in HV compared to LV rats (Figure 2A). Moreover, a greater amount of carbonylated proteins, a sign of oxidative damage to proteins, was also detected in the liver of HV as compared to LV rats (Figure 2C). The oxidative condition of the liver of HV rats was accompanied by reduced activity of ROS-detoxifying enzymes such as SOD, GPx and catalase, whose activities were all reduced in HV as compared to LV rats (Figure 2B).

Thereafter, we determined whether hepatic oxidative stress of HV rats could be reduced by OEA treatment. We found that OEA decreased the level of H_2_O_2_ in the liver of HFD-fed rats. In particular, the H_2_O_2_ level reached values significantly lower than in the liver of both HP and HV rats, and like those measured in the LV group (Figure 2A). As result, both MDA and carbonylated protein levels decreased to values even lower than LV values (Figure 2A,C).

Superoxide is converted into H_2_O_2_ by SOD and thereafter eliminated by antioxidant enzymes such as catalase, and GPx. We found that SOD and CAT activities were significantly increased, compared to HV rats, after OEA administration (Figure 2B). Although GPx activity had a trend toward an increase, after OEA treatment, the increase was not statistically significant.

Moreover, the antioxidant effect of OEA was also demonstrated in cultured hepatic cells, in which treatment with a mix of fatty acids (palmitate-oleate, 1:2) can induce hepatic oxidative stress [44]. We found that fatty acid-treated cells accumulated lipid droplets (Figure 2D) and signs of oxidative stress, such as significantly higher H_2_O_2_ levels, as compared to non-treated cells (Figure 2D). Moreover, SOD activity was significantly lower in fatty acid-treated cells compared to controls (Figure 2D). The addition of OEA to lipid-droplet enriched cells significantly decreased both lipid accumulation (Figure 2E) and H_2_O_2_ level with a concomitant significant increase in SOD activity (Figure 2D).

Taken together, these data suggest that OEA significantly reduces oxidative stress in the fatty liver by activating antioxidant pathways.

### 3.4. OEA Reduced the Endoplasmic Reticulum (ER) Stress

It is well known that ER stress triggers the unfolded protein response (UPR), which attempts to restore the normal ER homeostatic state within the cell. UPR signalling consists of three main branches, the Inositol Requiring Protein-1/X-box-Binding Protein 1 (IRE1/XBP1), the Activating Transcription Factor-6 (ATF6), and the Protein Kinase RNA (PKR)-like Endoplasmic Reticulum Kinase/eukaryotic Initiation Factor 2 alpha (PERK/eIF2α) [45,46,47,48,49,50]. Moreover, different enzymes and chaperons, among which BIP, are involved in the control of protein folding during ER stress.

**Figure 3:**
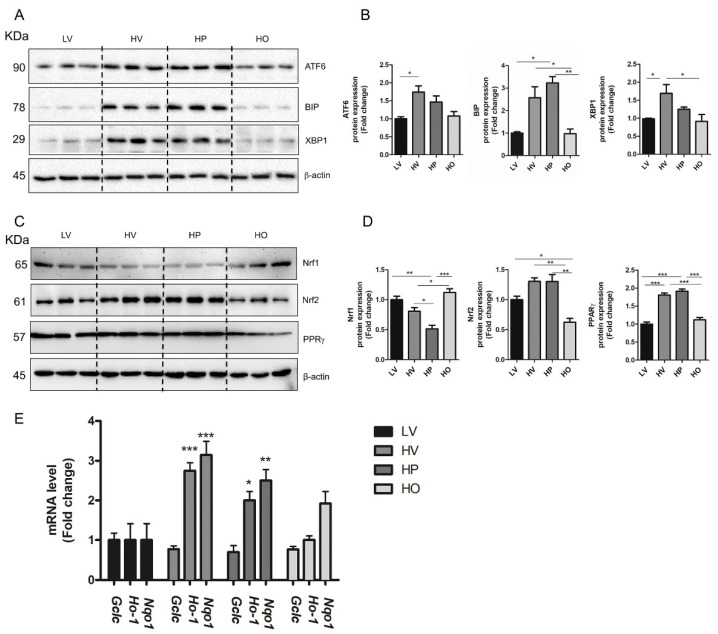
OEA reduces reticulum endoplasmic stress and differently affects Nrf1 and Nrf2 protein expression. (**A**) Representative immunoblots for Activating Transcription Factor 6 (ATF6), binding-immunoglobulin protein aka GRP-78 (BIP), and X-box-Binding Protein-1 (XBP1); β-actin was used as a loading control. (**B**) Quantification of protein expression. Values in the histogram were expressed as fold change relative to LV, the data represent the mean ± SEM (*n* = 3). (**C**) Representative immunoblots for nuclear factor erythroid-derived 2-related factor 1 (Nrf1), Nrf2 and peroxisome proliferator-activated receptor γ (PPARγ); β-actin was used as a loading control. (**D**) Quantification of protein expression reported as fold change relative to the control. Values in the histogram were expressed as fold change relative to the LV, the data represent the mean ± SEM (*n* = 3). (**E**) Glutamate-Cysteine Ligase Catalytic Subunit (*Gclc*), Heme oxygenase-1 (*Ho-1*) and NAD(P)H Quinone Dehydrogenase (*Nqo1*) mRNA expression in liver detected by *RT-qPCR*. Data are expressed as mean ± SEM (*n* = 4). * *p* < 0.05; ** *p* < 0.005; *** *p* < 0.001.

To examine whether OEA can affect HFD-induced ER stress, the protein level of XBP1, ATF6 and BIP in the liver of all groups of rats, was assessed by western blot (Figure 3A). The results showed that the expression of all these proteins was significantly increased in HV and HP, as compared to the LV group. Importantly, the expression of all these proteins was reduced in the HO group to values like those observed in LV rats (Figure 3A,B), thus suggesting that OEA can successfully abolish hepatic HFD-induced ER stress in rats.

### 3.5. OEA Differently Regulated the Expression of the Transcription Factors Nrf1 and Nrf2

Oxidative stress can modulate metabolic pathways by activating transcription factors, among which Nrf1 and Nrf2 play a key role [49,50,51,52,53,54,55]. We measured the protein expression of Nrf1, Nrf2, and PPARγ, this latter being an Nrf2-downstream transcriptional factor [56], involved in the regulation of lipid metabolism [34,57].

We found that the level of Nrf2 was increased in the liver of both HV and HP, compared to LV rats, and significantly decreased after OEA treatment (Figure 3C,D).

Considering that Nrf2 is a transcription factor that activates genes containing antioxidant response elements (ARE) in response to cellular stresses including ROS [35,36], the mRNA level of *Ho-1*, *Nqo1,* and *Gclc*, ARE-containing genes encoding for antioxidant enzymes, was investigated. As shown in Figure 3E, both *Ho-1* and *Nqo1* mRNA levels were significantly increased in HV and HP and decreased after OEA treatment compared to LV. On the contrary, no significant changes were observed for *Gclc* among groups.

We also found that the Nrf1 expression significantly decreased in the liver of HV and HP, as compared to LV rats, and was upregulated after OEA treatment reaching values significantly higher than those measured in both HV and HP (Figure 3D). Finally, we found that the protein level of PPARγ paralleled that of Nrf2, being higher in HV and HP vs. LV rats and significantly reduced by OEA treatment (Figure 3C,D).

Our data demonstrated that the anti-steatotic and antioxidant effects of OEA influence Nrf1 and Nrf2 with opposite mechanisms.

## 4. Discussion

In the present study, we reported that a two-week treatment with OEA to rats exposed to an HFD exerts anti-obesity effects and ameliorates both liver steatosis and hepatic stress, two conditions associated with diet-induced obesity. Moreover, OEA administration lowered hepatic fatty degeneration reducing hepatocellular damage.

The anti-steatotic effect of OEA in diet-induced NAFLD in rats has been already demonstrated and ascribed mostly to the promotion of fatty acid β-oxidation induced by the activation of PPARα [27]. With our study, we confirm that OEA treatment stimulates β-oxidation and, in addition, exerts inhibitory effects on the activities and expressions of key enzymes involved in fatty acid synthesis and transport, such as FAS, ACC, SCD1, and LDLR.

After OEA treatment we found reduced oxidative stress in obese animals, as detected by a decreased level of H_2_O_2_ and activation of key enzymes involved in the balance of the redox state, such as catalase, SOD, and GPx (Figure 2). Considering the role played by SOD and catalase in the protection of cells against oxidative damage [51,52] the increased activity of these enzymes following OEA treatment suggests a decreased hepatic oxidative stress in OEA-treated rats. The decreased hepatic oxidative state of OEA-treated rats ameliorated the oxidative damage as suggested by the reduction in the hepatic levels of carbonylated proteins and MDA (Figure 2), which represents the most prevalent biomarkers of protein and lipid peroxidation during liver injury [53,54]. Moreover, by in vitro experiment, a system that avoids systemic interferences, we were able to replicate the antioxidant effect of OEA against fat-induced oxidative stress (Figure 2D), thus confirming the observations made ex vivo.

It is noteworthy that all the antioxidant effects observed in the liver of the HO group, were significantly different from those of the HP group, thus indicating that OEA effects on the selected parameters might be specifically attributed to its direct pharmacological actions, rather than to its anorexigenic effect (Figure 2).

The ER plays an important role in the post-translational modifications and folding of proteins synthesized in the ribosomes [55]. One of the most abundant ER-resident proteins that increase the efficiency of protein folding of the nascent polypeptide is the Hsp70-type chaperone, BIP. Aberrant folding processes may lead to the accumulation of misfolded proteins, also known as ER stress. The unfolded protein response (UPR), a cell-signalling system that readjusts ER folding capacity to restore protein homeostasis, involves three signalling arms coordinated by IRE1-XBP1, PERK-eIF2a-ATF4, and ATF6 [52].

Liver fat causes the emergence of many complications such as ER stress [56]. ER stress markers are increased in patients with obesity-associated metabolic syndrome [57]. Both in vitro and in vivo studies have reported that ER stress, and the activation of the UPR signalling, play a critical role in the regulation of hepatic lipid metabolism [58]. Thus, the UPR pathway can activate lipogenic enzymes such as ACC2 and SCD1 and induce hepatic steatosis [59,60].

In our study, we showed that UPR activation, induced by HFD, was significantly reduced by OEA, as suggested by the lower expression of XBP1, ATF6 and BIP in HO vs. both HF and HP groups of rats (Figure 3A,B). To our knowledge, this is the first report showing a reduction of ER stress after OEA administration in a rat model of NAFLD. We can speculate that the OEA-induced anti-steatotic effect might relieve the pressure induced by lipid accumulation, thus ameliorating ER stress.

We found that OEA regulates the protein expression of Nrf1 and Nrf2 in opposite manners, i.e., increasing Nrf1 and decreasing Nrf2, as compared to both HP and HV groups (Figure 3C,D). Both observations are in accordance with the different roles recently attributed to these proteins. Nrf1 and Nrf2 are members of the cap’n’collar (CNC) basic-region leucine zipper (bZIP) family of proteins that play a crucial role in NAFLD and related oxidative stress response [61]. Moreover, these transcription factors are involved in the regulation of lipid metabolism [55,62]. It has been demonstrated that Nrf1 knockout mice accumulate lipids in the liver and exhibit a phenotype that mimics human non-alcoholic steatohepatitis. The somatic inactivation of Nrf1 expression leads to NAFLD and sensitizes hepatocytes to oxidative stress-induced cell toxicity and injury [63]. The lower expression of Nrf1 we observed in the steatotic liver of HV rats, as compared to LV rats, is coherent with these data reported in the literature. On the other hand, OEA-induced increase of Nrf1 expression might represent a novel mechanism by which OEA can regulate hepatic lipid metabolism in HFD-fed rats.

Nrf1, a protein largely localized in the cytoplasm, where it is anchored to the ER membrane [51], has been reported to regulate the proteasome system to ensure that misfolded proteins do not accumulate in the cell [64]. Although the role of Nrf1 in regulating the transcription of antioxidant genes is not yet fully understood, based on our results, we hypothesize a relationship between antioxidant enzyme activity and Nrf1 expression. This aspect will be elucidated in our future studies. However, considering the relationship between ER and Nrf1, with our data a possible post-translational control of antioxidant enzymes cannot be excluded.

Nrf2 is a transcription factor that plays a critical role in inflammation and antioxidant responses by regulating antioxidant and anti-inflammatory genes [53]. Indeed, Nrf2 is activated by ROS, inflammatory cytokines, and ER stresses [53]. Moreover, a role in the regulation of lipid metabolism and the occurrence of liver steatosis, has been also reported [55]. Regarding this latter aspect, conflicting data have been, so far, reported. Thus, while some studies reported that Nrf2 deletion leads to the onset of steatosis and progression of steatohepatitis [65,66,67,68], some others fail to demonstrate this effect and, on the contrary, reported hepatocytes triglyceride accumulation [55] and attenuation of hepatic steatosis [56] after Nrf2 activation. These apparent discrepancies could be due to the differences in the experimental model used to assay the Nrf2 effect.

In our experimental model of diet-induced obesity, we found that Nrf2 expression was increased upon HFD feeding (Figure 3D), probably due to the established high oxidative stress, and was decreased after OEA treatment.

Nrf2 is a transcription factor that, under normal conditions, is mainly localized in the cytoplasm through interaction with Kelch-like ECH-associated protein 1 (Keap1), its negative regulator, and is rapidly degraded by the ubiquitin-proteasome pathway [61].

During exposure to oxidative stress, oxidation of Keap1 cysteine residues leads to the release of Nrf2 from Keap1 inhibition and its translocation into the nucleus, where Nrf2 heterodimerizes with Maf and activates ARE containing genes [61]. Accordingly, in HV rats, in which a high level of oxidative stress was measured (Figure 2), we observed a higher mRNA expression of Nrf2 antioxidant target genes *Ho-1* and *Nqo1* compared to LV and HO (Figure 3E) thus supporting a potential protective role of Nrf2, as described [69]. However, despite the higher expression of Nrf2 in HV rats, compared to LV and HO, a lower activity of antioxidant enzymes SOD, catalase and GPx, was measured. Interestingly, Hardwick et al. reported that, during fatty liver progression, Nrf2 activation was associated with a decreased expression of antioxidant defense enzymes [70]. Therefore, we can hypothesize that although Nrf2 activation is prone to have a protective role in oxidative stress, the antioxidant defense, which resides in the activity of antioxidant enzymes, could fail due to multiple functional mechanisms beyond Nrf2. For instance, it was demonstrated that dietary lipids, which are easily peroxidized, can increase the accumulation of lipid peroxidation by-products (possibly aldehydes), resulting in decreased antioxidant capacity [71].

PPARγ is a regulator of lipid metabolism in hepatocytes; changes in the expression of this protein have been associated with non-alcoholic fatty liver diseases through the induction of lipogenic factors [57]. Recently, we demonstrated a PPARγ-mediated effect of OEA on reducing hepatic lipid accumulation in control rats [34]. Here, we found that HFD increased PPARγ expression (Figure 3D), and induced liver steatosis, both restored by OEA administration. Considering the role of PPARγ as a positive modulator of de novo lipogenic enzymes, the decreased expression we measured in the liver of OEA-treated animals is well-correlated with the lower activity and expression of ACC and FAS we measured in HO vs. HV rats. Based on recent findings reporting an Nrf2-mediated regulation of PPARγ expression in the modulation of hepatic lipid metabolism [72], and the coordinate modulation of these factors we measured, the relationship between Nrf2 and PPARγ in the liver of HFD-fed rats awaits further investigations.

## 5. Conclusions

In summary, our data demonstrated that OEA can reduce obesity and fatty liver and ameliorate parameters of oxidative stress and ER stress in the liver of HDF-fed rats.

Here, we highlighted a critical role for both Nrf1 and Nrf2 in OEA-induced antioxidant and antisteatotic effects. However, while Nrf1 seems to be coherent with the antioxidant pathway, Nrf2 plays a major role in regulating lipid metabolism. Although these results pave the way to further investigations, we propose new molecular targets to be considered in the beneficial effects of OEA in the liver.

Lastly, considering the important role played by dietary fatty acids on oxidative stress and liver steatosis, as well as their impact on the endogenous levels of OEA and other acylethanolamides, it will be important to explore in the future how a different composition in dietary fatty acids might influence OEA effects on those parameters that were investigated in the present study.

## Data Availability

Data is contained within the article.
